# Hemoglobin profile and molecular characteristics of the complex interaction of hemoglobin Doi-Saket [α9(A7) asn > lys, HBA2:c.30C > a], a novel α2α1 hybrid globin variant, with hemoglobin E [β26(B8) Glu > lys, HBB:c.79G > A] and deletional α^+^-thalassemia in a Thai family

**DOI:** 10.1080/07853890.2023.2264174

**Published:** 2023-10-05

**Authors:** Sitthichai Panyasai, Kunyakan Khongthai, Surada Satthakarn

**Affiliations:** a Department of Medical Technology, School of Allied Health Sciences, University of Phayao, Phayao, Thailand; b Health Promoting Hospital, Chiang-Mai, Thailand

**Keywords:** Hb Doi-Saket, α-globin variant, α^+^-thalassemia, HbE, Thailand

## Abstract

**Background:**

An increasing number of α-hemoglobin (Hb) variants is causing various clinical symptoms; therefore, accurate identification of these Hb variants is important.

**Objective:**

This study aimed to describe the molecular and hematological characteristics of novel Hb Doi-Saket that gives rise to a typical α^+^-thalassemia phenotype in carriers with and without other hemoglobinopathies.

**Materials and Methods:**

Biological samples from a proband and his family members were analyzed. Hematological profiles were analyzed using a standard automated cell counter. Hb was analyzed by capillary electrophoresis and high-performance liquid chromatography. Mutations and globin haplotype were identified by DNA analysis. Novel diagnostic tools based on allele-specific polymerase chain reaction (PCR) and PCR–restriction fragment length polymorphism were developed.

**Results:**

Hb analysis showed a major abnormal Hb fraction, moving slower than HbA, and a minor Hb fraction alongside HbA_2_ in the proband, his father, and son. DNA analysis of the α-globin gene identified the -α^3.7^ deletion and in *cis* the C > A mutation on codon 9 of the α2α1 gene, corresponding to Hb Doi-Saket [α9(A7) Asn > Lys]. This mutation could be identified using newly developed allele-specific PCR-based assays. The Hb Doi-Saket al.lele was significantly associated with haplotype [- + M + + 0 -]. Interaction of α^Doi-Saket^ with β^E^ globin chains led to a new Hb variant (HbE Doi-Saket). Phenotypic expression was clinically silent in heterozygotes and might present slight microcytosis.

**Conclusions:**

Hb Doi-Saket emphasizes a great diversity present in α-globin gene. The mutation in this family from Thailand was linked to -α^3.7^ and caused mild microcytosis in the carriers. The combination of this variant with deletions in α genes might cause a severe clinical phenotype. Different methods of separation can provide useful information in diagnosis, and a complete molecular approach is needed for confirmation before considering patient management.

## KEY MESSAGES

1. The Hb Doi-Saket is a novel α-globin variant mutation occurring in the α2-globin gene in *cis* to the -α^3.7^ kb chromosome.
2. The carrier of Hb Doi-Saket may present slight microcytosis and have severe clinical entities when it interacts with deletions in α-globin genes.
3. Hb analysis with the HPLC system could completely separate Hb Doi-Saket and its derivative from other Hbs.

## Introduction

Thalassemia and hemoglobinopathies are monogenic disorders, which are commonly found in Southeast Asian countries, including Thailand, where the population (up to 40%) carries a potentially significant mutation in hemoglobin (Hb) [1]. In addition to HbE [β26(B8) Glu > Lys, HBB:c.79G > A] and Hb Constant Spring (HbCS) [α_2_142, Stop > Gln, HBA2:c.427T > C], which are relatively common in Thailand, several other abnormal Hb resulting from mutations of α and β genes have occasionally been reported [2]. Approximately 60 human Hb variants have been characterized among Thai individuals [3,4]. β-Hb variants are most commonly detected, whereas α-Hb variants are infrequent. δ-globin mutation is also rare [5–7], and no mutation of the α2α1 hybrid gene has been documented yet in Thai individuals. The silent variant Hb G-Philadelphia [α2 or α1 68(E17) Asn > Lys, HBA2:c.207C > G (or HBA1) or 207 C > A] has been identified in the α2α1 hybrid gene in *cis* to the -α^3.7^ chromosome. This association is reported to be relatively common in the Black African ancestry [8]. In addition, C > A mutation has been characterized at the same position on the α2 gene, and both C > G and C > A mutations result in the replacement of an asparagine residue with lysine. Microcytosis and hypochromia may be characterized, and it is consistently associated with the (-α^G-Philadelphia^/-α) genotype. The clinical HbH phenotype is observed when it is associated with deletions in the α genes [9]. The circulating level of Hb G-Philadelphia is different depending on the occurrence of mutation on double- and single- locus chromosomes with approximately 30–35% in heterozygotes with C > G mutation on the α2α1 hybrid gene and 25–27% in those with C > A mutation on the α2 gene [10]. Several variations of Hb G-Philadelphia exist in the α-globin gene; therefore, assessing whether a mutation that characterizes a variant chain has occurred on the α2- or α1-globin gene or α2α1 hybrid gene is difficult. If another α-Hb variant shows a similar pattern of migration or elution, making the identification difficult, it could be underestimated if a single characterization method is employed for diagnosis.

In this study, we characterized a novel α-Hb variant, Hb Doi-Saket, a mutation present in the α2α1 hybrid gene in a Thai family. Furthermore, we described the complex interaction of this variant with heterozygous HbE.

## Materials and methods

### Subjects and hematological analysis

The proband, a 31-year-old Thai man, his father, mother, wife, and son were recruited after an anomalous Hb fraction was observed in the proband during a thalassemia diagnosis program among married couples. After informed consent was obtained, peripheral blood specimens were collected and sent to the School of Allied Health Sciences, University of Phayao for further analysis of the unknown Hb variant. Hematological parameters were assessed using a UniCel DxH 800 automated blood cell counter (Beckman-Coulter Co., Miami, FL, USA), and parameters related to iron metabolism [serum iron, serum ferritin, total iron binding capacity, and transferrin saturation were determined using an automated immunoassay analyzer (ARCHITECT i2000SR, Abbott Laboratories, Abbott Park, IL, USA). Hb was analyzed using capillary electrophoresis (CE; Capillarys 2 Flex Piercing, Sebia SA, Lisses, France) and two automated cation-exchange high-performance liquid chromatography (HPLC) systems: a Premier Resolution system (Trinity Biotech, Bray, Country Wicklow, Ireland) using the high-resolution mode and the VARIANT II system (Bio-Rad Laboratories, Hercules, CA, USA) using the β-Thalassemia Short Program. The study protocol was approved by the institutional review board at the University of Phayao, Thailand (UP-HEC 1.3/020/64). Written informed consent obtained from all participants.

### Consent to publish

Consent to publish patient and study details was obtained from all participants included in this study.

### DNA analysis

Genomic DNA was extracted from leukocytes using a genomic DNA isolation kit (BIO-HELIX Co., LTD., Keelung city, Taiwan). The six common α-thalassemia, including α-thalassemia-1 (SEA and THAI deletions), deletional α^+^-thalassemia (-α^3.7^ and -α^4.2^), Hb Constant Spring (α^CS^), and Hb Paksé (α^Paksé^) mutations, was analyzed by gap-PCR and allele-specific PCR as previously described [11–13]. HbE and Hb Q-Thailand [α1 74(EF3) Asp > His; HBA1:c.223G > C] had the same retention time in HPLC and same migration zone on the electropherogram compared with those of the novel Hb variant described herein, which has been documented in Thai population using allele-specific PCR as previously described [14,15]. The α1- and α2-globin genes were amplified using the primers C1 (5′-TGGAGGGTGGAGACGTCCTG-3′) and B (5′-GAGGCCCAAGGGGCAAGAAGCAT-3′) [12] and C1 and C3 (5′-CCATTGTTGGCACATTCCGG-3′) [16], respectively. The hybrid -α^3.7^ gene was amplified using the primers A (5′-CCCAGAGCCAAGTTTGTTTATCTGT-3′) and B. The amplification products were purified using a GF-1 AmbiClean kit (Vivantis Technologies, Selangor Darul Ehsan, Malaysia) according to the manufacturer’s instructions. The amplified α-globin genes were then directly sequenced using ABI Prism 3130 XL (Applied Biosystems Life Technologies, Carlsbad, CA, USA). Haplotyping of the α-globin gene was carried out using seven most common polymorphisms, consisting of one broadly triallelic inter ζ-globin hypervariable region (HVR) and six restriction fragment length polymorphisms (RFLPs) including *Xba*I site of the 5′ ζ2-globin gene, *Sac*I site of the 3′ ζ2-globin gene, *Acc*I site of the 3′ ψα2-globin gene, *Rsa*I site of the 5′ α2-globin gene, and *Pst*I sites of the 5′ α1- and 5′ θ1-globin genes. The RFLP region and inter ζ-globin HVR region were amplified as previously described [17]. Haplotypes were designed by determining the presence or absence of cleavage at each site and by compiling the results as one pattern.

### Identification of Hb Doi-Saket by PCR–RFLP

A C > A substitution at the third nucleotide of codon 9 on the α2α1 hybrid gene, which is responsible for Hb Doi-Saket lacking a HpyCH4IV restriction site, was confirmed by PCR–RFLP analysis. A 975-bp fragment specific for the α1-globin gene was amplified using specific primers C1 and B. The PCR mixture (50 µL) contained 100–200 ng genomic DNA, 60 pM of primers C1 and B each, 200 μM dNTP, 1.5 mM MgCl_2_, 1 M betaine, 5% dimethyl sulfoxide (DMSO), and 1.5 U *Taq* DNA polymerase (Vivantis Technologies) in 10 mM Tris-HCl buffer (pH 9.1) containing 50 mM KCl and 0.1% Triton X-100. Amplification was performed using a thermal cycler (Cyclerus personalis; Bio-Rad Laboratories). An initial denaturation step at 94 °C for 3 min was followed by 10 cycles at 94 °C for 30 s, 60 °C for 30 s, and 68 °C for 2 min, 20 cycles at 94 °C for 30 s, 60 °C for 30 s, and 68 °C for 2 min, and additional 20 sec in each cycle. The amplified fragment was completely digested with HypCH4IV (5′-A^▼^CGT-3′) (New England Biolabs, Inc., Beverly, MA, USA), analyzed by 2% agarose gel electrophoresis, and visualized under ultraviolet (UV) light after staining the gel with ethidium bromide. The Hb Doi-Saket-derived fragment was 561-bp long, whereas its normal counterpart with a HpyCH4IV restriction site at codon 9 is digested to 306- and 255-bp fragments. To assess whether nucleotide transition has occurred on the chromosome with the deletion or those with the normal α2 and α1 globin genes, the α2 globin gene was amplified using specific primers C1 and C3, and a 1,080-bp fragment specific for this gene was digested using HypCH4IV under the same conditions.

### Simultaneous identification of Hb Doi-Saket and Hb Q-Thailand using a newly multiplex allele-specific PCR (ASPCR)

To provide a method for simple and rapid diagnosis, multiplex ASPCR for simultaneous detection of Hb Doi-Saket and Hb Q-Thailand was developed. We designed the forward primer SP42 (5′-TGTCTCCTGCCGACAAGACCAAA-3′), located at 5′ upstream and specific for C > A mutation, which was used with primer B, located at 3′ upstream, to produce a 729-bp fragment specific for the α^Doi-Saket^ allele, and primers αG20 and B were used to generate a specific 416-bp fragment of the α^Q-Thailand^ allele as previously described [14]. The internal control fragment (199 bp) of PCR amplification was generated using primers SP44 (5′-CGGCCCCACTGACCCTCTTCTCT-3′) and B. The reaction mixture (50 µL) contained 100–200 ng genomic DNA, 15 pM of primers αG20 and SP44 each, 18 pM primer SP42, 45 pM primer B, 200 μM dNTP, 1.5 mM MgCl_2_, 5% DMSO, 1 M betaine, and 1.0 U of *Taq* DNA polymerase (Vivantis Technologies) in 10 mM Tris-HCl buffer (pH 9.1) containing 50 mM KCl and 0.1% Triton X-100. Amplification was carried out on a thermal cycler (Cyclerus personalis). An initial denaturation step at 94 °C for 3 min was followed by 30 cycles at 94 °C for 30 s, 62 °C for 30 s, and 72 °C for 1 min, and a final extension at 72 °C for 10 min. The amplified product was analyzed by electrophoresis using a 1.5% agarose gel and visualized using UV light after staining the gel using ethidium bromide.

## Results

**Figure 1.**
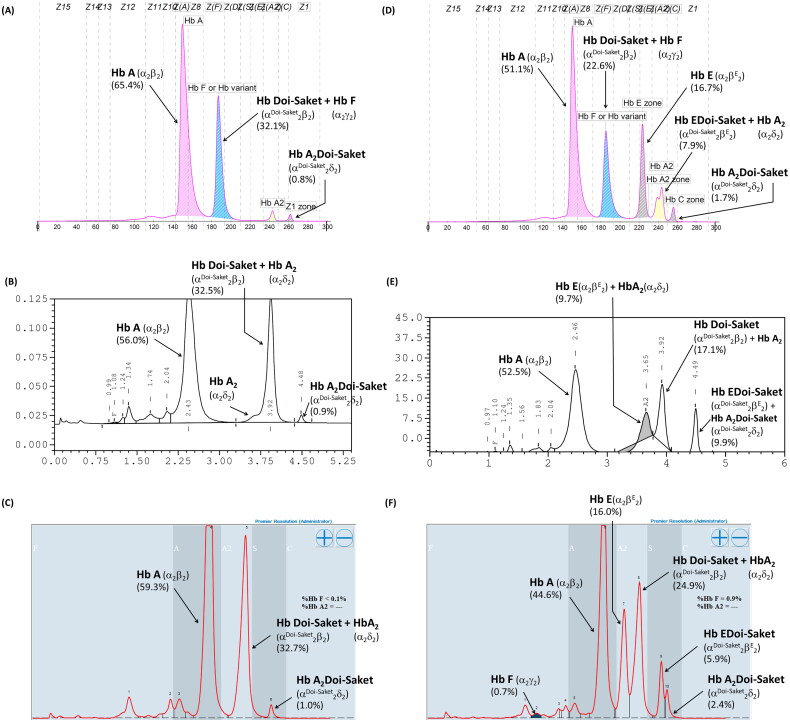
Hb analysis of simple heterozygosity of Hb Doi-Saket identified in the proband (A–C) and double heterozygosity of Hb Doi-Saket and HbE identified in his son (D–F). A and D show the Electropherograms; Hb Doi-Saket concealed HbF at electrophoretic zone 7. B and E are HPLC chromatograms analyzed using the VARIANT II system; Hb Doi-Saket was eluted by overlapping with HbA_2_ with a retention time of 3.92 min. C and F represent the HPLC chromatograms obtained using the Premier Resolution system; Hb Doi-Saket completely overlapped with HbA_2_ during elution.

Hematological parameters, iron profile, and genotypes of the proband and his family members are detailed in Table 1. The father and mother, who were 64- and 58-year-old, respectively, had no anemia or abnormalities in red cell parameters. The wife and son had no anemia, but their mean corpuscular volume (MCV) and mean corpuscular hemoglobin (MCH) values were slightly low. The parameters observed in the proband revealed no anemia and slightly low MCV and MCH. Their microcytic hypochromic red blood cells might be affected by thalassemia. CE revealed a fraction of HbA and HbA_2_ accounting for 2.9% of the total Hb, which is usually observed in a normal individual, as was observed in the mother. HbA and HbE fractions accounted for 29.7% of the total Hb were observed in the wife, which indicated simple heterozygous HbE. Hb analysis in the proband and his father revealed fractions of HbA, HbA_2_, and HbF and a small Hb fraction at zone 1, with HbF accounting for 32.1% and 31.6% of the total Hb in the proband and father, respectively (Figure 1A). With this result, they should be initially diagnosed with either β-thalassemia or hereditary persistence of fetal hemoglobin owing to elevated HbF levels. The presence of a minor HbA_2_ fraction on the electropherogram indicated a defective δ-globin gene. In Figure 1D, the electropherogram presents HbA, HbF, and HbE and two additional anomalous Hb fractions at the migration zone of A_2_ and C, accounting for a small proportion of the total Hb in the eight-year-old son. This indicated that the boy probably had β-thalassemia/HbE disease, together with an unknown Hb variant. Hb analysis using VARIANT II HPLC did not show elevated HbF, while major peaks of HbA and anomalous Hb, eluted partially overlapping HbA_2_ at a retention time of 3.92 min, represented approximately 31.2–32.5% of the total Hb, and a minor Hb peak at a retention time of 4.48 min representing 0.9% of the total Hb were observed in both the father and proband (Figure 1B). For the son, an HbE peak was consistently observed, which was coeluted with HbA_2_ at the retention time of 3.65 min and accounting for 9.7% of the total Hb; however, the apparent HbF peak did not appear in the chromatogram. Additionally, two unknown and abnormal Hb peaks, eluted at retention times of 3.92 and 4.49 min, were also observed on the chromatogram, with the latter having the same retention time as that detected for the proband and his father (Figure 1E). Hb analysis using Premier Resolution HPLC showed a major peak of HbA, and two additional abnormal Hb peaks were observed in the father and proband (Figure 1C). One of these peaks eluted overlapping HbA_2_ completely at a retention time of 5.835 and 5.853 min in the father and proband and represented approximately 32.0% and 32.7% of the total Hb, respectively. The other peak eluted at the retention time of 6.178 and 6.181 min in the father and proband, respectively. Premier Resolution HPLC revealed a major peak of HbA accounting for 44.6% of the total Hb and HbE, which eluted at the retention time of 5.509 min, accounting for 16.0% of the total Hb in the son. Moreover, three additional abnormal Hb peaks were observed (Figure 1F), and two of them had the same retention time (5.810 and 6.188 min) as those detected in the proband and his father. The other abnormal peak eluted at the retention time of 6.090 min, accounting for 5.9% of the total Hb. The Hb profile of the proband’s mother and wife using Premier Resolution HPLC revealed A_2_A and EA, respectively. Anomalous Hb peaks were not observed. The amount of HbE in the wife was 31.5% of the total Hb (Table 1), indicating that she was heterozygous for HbE.

**Table 1.** Hematological data and globin genotypes of the proband and his family members.

Parameters	Father	Mother	Proband	Wife	Son
Age (years)	64	58	31	33	8
RBC count (x10^12^/L)	5.51	4.18	6.16	5.92	4.79
Hb (g/dL)	15.2	12.0	16.2	13.7	12.3
Hct (L/L)	46	36.7	49.1	41.4	38.1
MCV (fL)	83.5	87.6	79.7	70.0	79.6
MCH (pg)	27.6	28.8	26.4	23.2	25.6
MCHC (g/dL)	33.1	32.8	33.1	33.1	32.2
RDW-CV (%)	14.8	13.9	13.9	14.1	13.7
CE-Hb Profile^a^	A_2_A with Hb Doi-Saket	A_2_A	A_2_A with Hb Doi-Saket	EA	EA with Hb Doi-Saket
Hb A (%)^a^	65.8	97.1	65.4	70.1	51.1
Hb A_2_ (%)^a^	1.8	2.9	1.7	4.0	7.9*
Hb E (%)^a^	0	0	0	29.7	16.7
Hb F + Hb Doi-Saket (%)^a^	31.6	0	32.1	0.2	22.6
Hb A_2_Doi-Saket (%)^a^	0.8	0	0.8	0	1.7
Premier HPLC-Hb Profile^b^	A with Hb Doi-Saket	A_2_A	A with Hb Doi-Saket	EA	EA with Hb Doi-Saket
Hb A (%)^b^	59.6	89.8	59.3	60.4	44.6
Hb A_2_+E (%)^b^	0	3.3	0	31.5	16.0
Hb F (%)^b^	0	0	0	1.9	0.7
Hb A_2_+Hb Doi-Saket (%)^b^	32.0	0	32.7	0	24.9
Hb EDoi-Saket (%)^b^	0	0	0	0	5.9
Hb A_2_Doi-Saket (%)^b^	1.0	0	1.0	0	2.4
Variant II HPLC-Hb Profile^c^	A with Hb Doi-Saket	not done	A with Hb Doi-Saket	not done	EA with Hb Doi-Saket
Hb A (%)^c^	56.6	not done	56.0	not done	52.5
Hb A_2_+E (%)^c^	0	not done	0	not done	9.7
Hb F (%)^c^	0.4	not done	0.2	not done	1.2
Hb A_2_+Hb Doi-Saket (%)^c^	31.2	not done	32.5	not done	17.1
Hb A_2_Doi-Saket (%)^c^	0.9	not done	0.9	not done	9.9**
Iron profile					
Ferritin (µg/L)	120.5	not done	74.2	not done	not done
Serum iron (µg/dL)	65.3	not done	130.8	not done	not done
TIBC (µg/dL)	262.9	not done	349.4	not done	not done
UIBC (µg/dL)	197.6	not done	218.6	not done	not done
%Tsat (%)	24.8	not done	37.4	not done	not done
α-globin genotype	-α^Doi-Saket(3.7)^/αα	αα/αα	-α^Doi-Saket(3.7)^/αα	αα/αα	-α^Doi-Saket(3.7)^/αα
β-globin genotype	β^A^/β^A^	β^A^/β^A^	β^A^/β^A^	β^E^/β^A^	β^E^/β^A^

RBC = Red Blood Cells; Hb = Hemoglobin; Hct = Hematocrit; MCV = Mean Corpuscular Volume; MCH = Mean Corpuscular Hemoglobin; RDW-CV = Coefficient of Variation of the Red Cell Distribution Width; Tsat = Transferrin saturation.

^a^ Determined using Capillary Electrophoresis, Capillarys 2.

^b^ Determined using High Performance Liquid Chromatography, Premier Resolution, Trinity Biotech.

^c^ Determined using High Performance Liquid Chromatography, β-thal short program, Bio-Rad variant II system.

* The percentage included HbA_2_ and HbEDoi-Saket.

** The percentage included HbA_2_Doi-Saket and HbEDoi-Saket.

**Figure 2.**
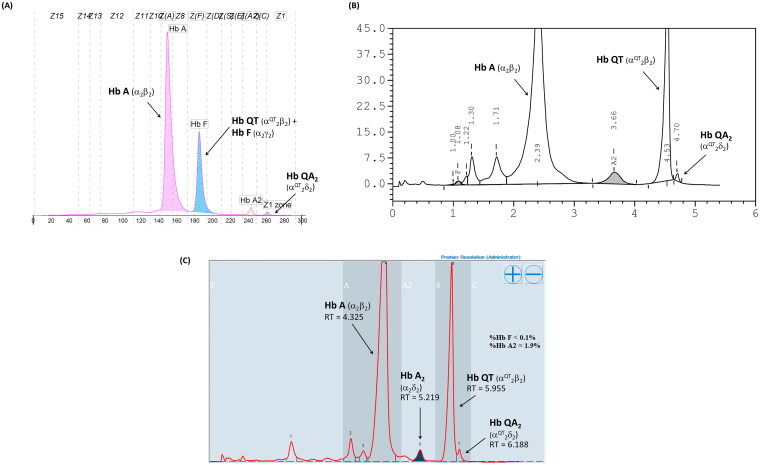
Hb Separation profiles of the heterozygous Hb Q-Thailand using (A) capillary electrophoresis, (B) VARIANT II HPLC, and (C) Premier Resolution HPLC.

**Figure 3.**
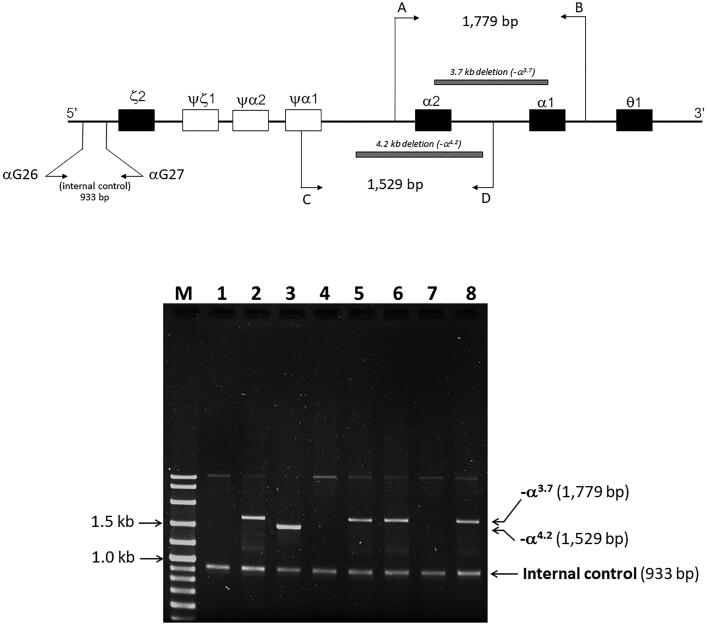
Locations of the four primers, the breakpoints of -α^3.7^ and -α^4.2^ deletions, and amplified DNA fragment. The 1,529- and 1,779-bp fragments are specific for -α^3.7^ and -α^4.2^, respectively. M, 100-bp marker; lane 1; normal control; lane 2, -α^3.7^ carrier; lane 3, -α^4.2^ carrier; lane 4, mother; lane 5, father; lane 6, proband; lane 7, wife of proband; and lane 8, son of proband.

**Figure 4.**
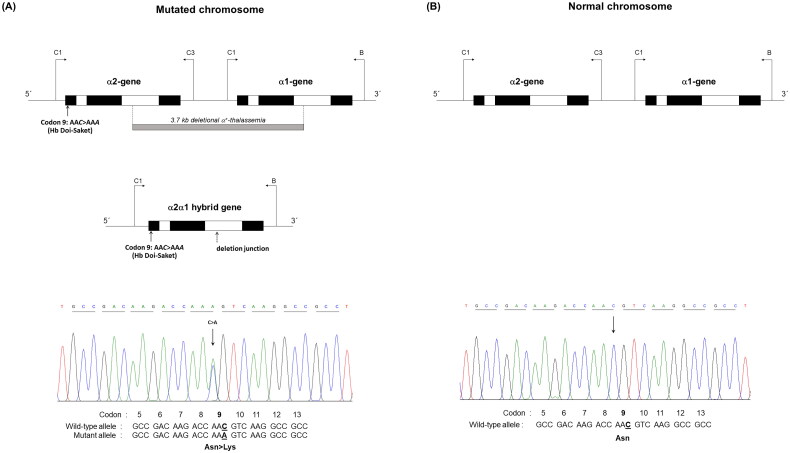
(A) the locus chromosome with -α^3.7^ deletion and forward directional nucleotide sequence obtained from the α2α1 hybrid and α1 genes. (B) the lack of deletion on the locus chromosome and forward directional nucleotide sequence obtained from the α2 gene. The arrow indicates replacement of the heterozygote nucleotide (codon 9 AA*C*>AA*A*), and any identical nucleotide substitution in the same place was not characterized in the α2 gene.

**Figure 5.**
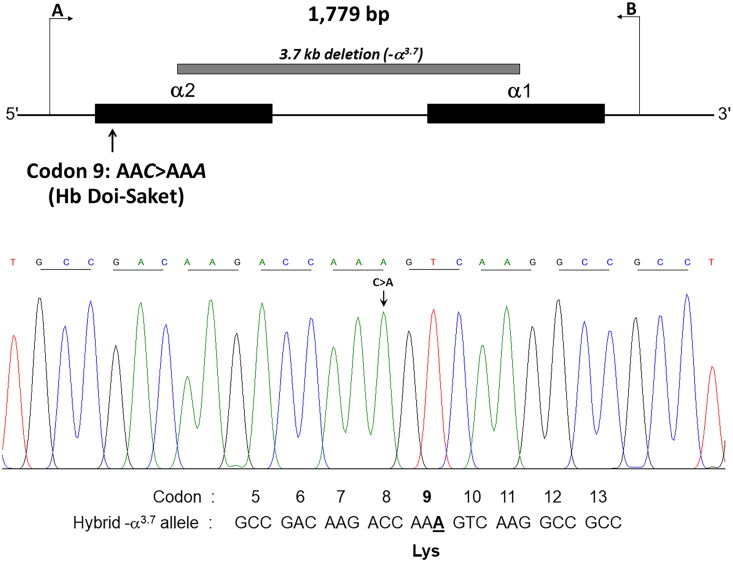
The hybrid -α^3.7^ gene and forward directional nucleotide sequence obtained from this gene. The arrow indicates nucleotide replacement.

**Figure 6.**
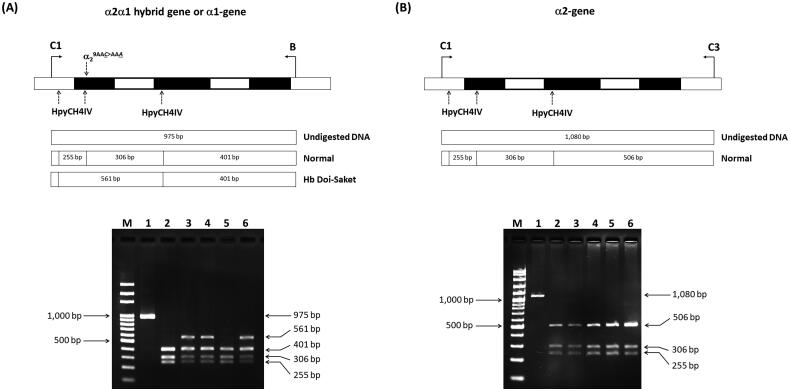
Identification of AA*C*>AA*A* mutation by HpyCH4IV digestion of the PCR product. (A) HpyCH4IV digestion of the in *cis* α2α1 hybrid and α1 genes. Lane 1 is the undigested amplified DNA (975 bp). lanes 2 and 5 are HpyCH4IV-digested amplified DNA of the proband’s mother and wife, who had normal α-globin allele (αα/αα). the 561-bp digested fragment in lanes 3, 4, and 6 indicates the presence of Hb Doi-Saket mutation in the proband, his father, and son. (B) HpyCH4IV digestion of the α2 gene. Three fragments of 506, 306, and 255 bp were produced, whereas the 561-bp fragment was not produced owing to the absence of AA*C*>AA*A* mutation.

Given that the electrophoretic migration of anomalous Hb variant (Figure 1A) is quite similar to that of Hb Q-Thailand (Figure 2A), which is occasionally found in the population of Southeast Asia, an allele-specific PCR for Hb Q-Thailand was carried out as previously described [14], which showed a negative result for whom this variant was observed. This method identified the β^E^ allele in the wife and son but not in the proband and his parents, indicating that his wife and son were carriers of HbE. The appearance of a major anomalous Hb fraction alongside a minor Hb fraction in both HPLC and CE analyses suggests the possibility of structural abnormality in the α-globin chain. DNA analysis indicated the -α^3.7^, the most common type of α^+^-thalassemia in the proband, his father, and son; however, no abnormality of the α-globin gene was observed in the mother and wife (Figure 3). Therefore -α^3.7^ observed in the proband was inherited from his father. Sequence analysis of the α-globin genes on both chromosomes (Figure 4A and B) reveled AA*C*>AA*A* transition in the heterozygous state at codon 9, which resulted in the replacement of asparagine by lysine in the proband, his father, and son who was a carrier of the -α^3.7^ (Figure 4A). This result indicated that AA*C*>AA*A* transition observed in the proband was certainly inherited from his father. In addition, sequence analysis of the hybrid -α^3.7^ gene in the proband, his father, and son revealed an A at the third nucleotide of codon 9 (Figure 5). This confirmed that the C > A transition was associated with the -α^3.7^ gene. Based on this result, we could assess the C > A transition at codon 9, which characterizes a variant chain that occurs on the first exon of the α2α1 hybrid gene; this mutation has not yet been documented in the HbVar database [18]. We named this Hb variant as Hb Doi-Saket after the name of the place where the patient lived. The AA*C* to AA*A* mutation at codon 9 led to the lack of HpyCH4IV restriction site (5′-A^▼^CGT-3′) on the α-gene; this mutation was confirmed by PCR–RFLP analysis (Figure 6). The 561-bp digested fragment, specific for Hb Doi-Saket, was observed either on the α2α1 hybrid gene or α1 gene of the proband, his father, and son (Figure 6A); however, it was not observed on the α2 gene (Figure 6B). Consequently, the results of DNA sequencing and the PCR–RFLP analysis confirmed that they were carriers of Hb Doi-Saket, linked in *cis* to the -α^3.7^ chromosome. Based on these results, we concluded that the proband and his father were heterozygous for Hb Doi-Saket and -α^3.7^ with genotype -α^Doi-Saket(3.7)^/αα, and the son was double heterozygous for these mutations and HbE.

**Figure 7.**
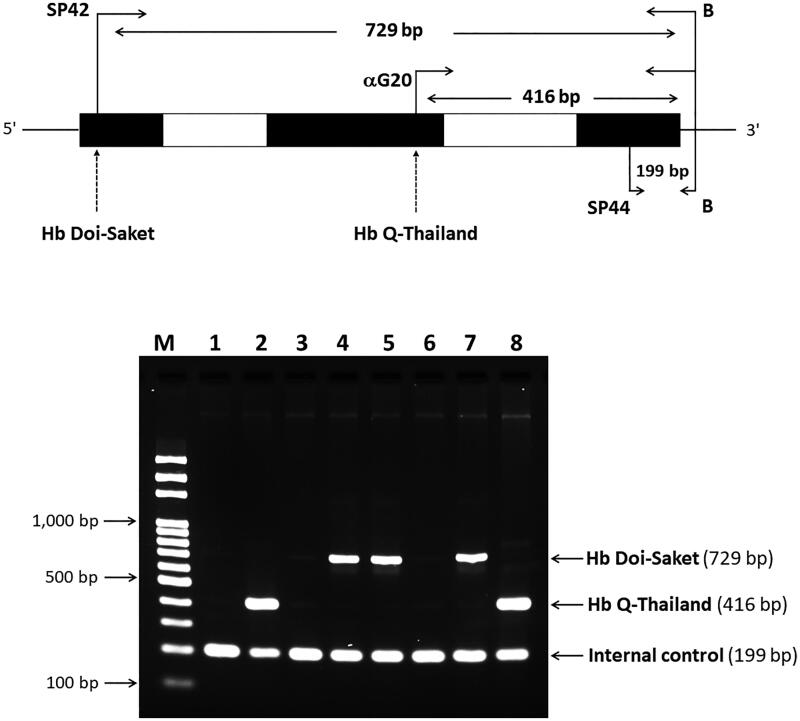
A multiplex ASPCR Assay for simultaneous identification of Hb Doi-Saket and Hb Q-Thailand. The locations and orientations of primers are indicated. The 729-bp fragment generated using SP42 and B is specific for α^Doi-Saket^, whereas the 416-bp fragment generated using αG20 and B is specific for α^Q-Thailand^. The 199-bp fragment is an internal control generated using SP44 and B. M, 100-bp ladder; lane 1, normal DNA; lane 2, DNA control of Hb Q-Thailand; lane 3, mother; lane 4, father; lane 5, proband; lane 6, wife of proband; lane 7, son of proband; and lane 8, Hb Q-Thailand carrier.

After establishing a multiplex allele-specific PCR method for simultaneous, rapid, and differential diagnosis of Hb Doi-Saket and Hb Q-Thailand, a 729-bp amplified fragment specific for Hb Doi-Saket allele was clearly observed in the carriers of Hb Doi-Saket (Figure 6, lanes 4,5, and 7), while carriers of Hb Q-Thailand showed the 416-bp fragment (Figure 7, lanes 2 and 8). A 199-bp fragment, specific for the normal allele, was observed in all subjects (Figure 7). Therefore, normal DNA control and normal individuals showed only one fragment of 199 bp (Figure 7, lanes 1, 3, and 6). The results of α-globin gene haplotype analysis are summarized in Table 2. The identified Hb Doi-Saket variant was linked with -α^3.7^ deletion within the 3′ untranslated region of the α2 gene to IVSII of the α1 gene, covering the α*Pst*I haplotype [17,19]; only the α2α1 hybrid gene, α^Doi-Saket^, remained intact in the mutated chromosome. The restriction fragment of the α*Pst*I site was absent on the -α^3.7^ chromosome. Digestion with *Pst*I at this position, showed the hemizygous pattern [-/0] owing to -α^3.7^ deletion (Table 2). The haplotype α-globin gene was also determined in all four family members of the proband, leading to successful completion of haplotype segregation. This result demonstrated that the Hb Doi-Saket allele was strongly associated with the α-globin gene haplotype [- + M + + 0 -].

**Table 2.** α-Globin gene haplotypes associated with Hb Doi-Saket.

		**α-globin haplotype (5′>3′)**														
		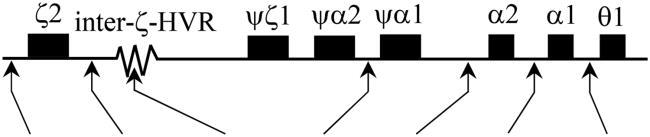							**Hb Doi-Saket (-α^Doi-Saket^) linked α-haplotype**							
**Subjects**	**α-genotype**	*Xba* I	*Sac* I	[S/M/L]	*Acc* I	*Rsa* I	α*Pst* I	θ*Pst* I								
Father (I-1)	-α^Doi-Saket^/αα	[-/-]	[+/-]	[M]	[+/-]	[+/-]	[-/0]	[-/-]	**-α^Doi-Saket^**	**[ -**	**+**	**M**	**+**	**+**	**0**	**- ]**
									αα	[ -	–	M	–	–	–	- ]
Mother (I-2)	αα/αα	[-/-]	[+/+]	[L]	[+/+]	[-/-]	[-/-]	[-/-]	αα	[ -	+	L	+	–	–	- ]
									αα	[ -	+	L	+	–	–	- ]
Proband (II-1)	-α^Doi-Saket^/αα	[-/-]	[+/+]	[L/M]	[+/+]	[+/-]	[-/0]	[-/-]	**-α^Doi-Saket^**	**[ -**	**+**	**M**	**+**	**+**	**0**	**- ]**
									αα	[ -	+	L	+	–	–	- ]
Wife (II-2)	αα/αα	[+/-]	[+/+]	[L/M]	[+/+]	[+/-]	[-/-]	[-/-]	αα	[ -	+	L	+	–	–	- ]
									αα	[ +	+	M	+	+	–	- ]
Son (III-1)	-α^Doi-Saket^/αα	[+/-]	[+/+]	[M]	[+/+]	[+/+]	[-/0]	[-/-]	**-α^Doi-Saket^**	**[ -**	**+**	**M**	**+**	**+**	**0**	**- ]**
									αα	[ +	+	M	+	+	–	- ]

+ and - represent the presence and absence of the restriction enzyme sites, respectively. 0 indicates deletion. S and M indicate inter ζ hyper variable region (HVR).

## Discussion

More than 1,800 Hb variants have been documented in the HbVar database, 600 of which are α-Hb variants [18]. Most of them are separated from normal Hb by routine Hb analysis using electrophoresis or HPLC; however, α-Hb variants concealed within normal Hb or with similar migration or elution patterns lead to underestimation of certain variants. Here we report a novel α-Hb variant, Hb Doi-Saket that was initially identified incorrectly as Hb Q-Thailand. Hb Doi-Saket was characterized by a C > A substitution at the third base of codon 9 of the α2-globin gene leading to the replacement of asparagine with a lysine residue. A heterozygosity of this mutation was identified in the proband, his father, and son. Interestingly, further analysis of the α-globin gene exhibited the -α^3.7^ deletion, the most common type of α-thalassemia [20] in the proband, his father, and son, while this was not observed in his mother and wife. This result clearly indicated that the -α^3.7^ and the α^Doi-Saket^ mutations are linked together on the same chromosome [-α^Doi-Saket(3.7)^]. Hematological analysis showed no anemia in subjects heterozygous for this variant, suggesting that the functional properties of Hb Doi-Saket and its derivative are similar to those of HbA. This could be explained by the fact that the mutated residue lies inside the helix A and does not take part in the α/β contact or is not involved in the heme pocket [21]. Therefore, no detrimental effects should be expected in individuals carrying Hb Doi-Saket. Slightly low MCV and MCH observed in the proband and his son, who coinherited Hb Doi-Saket with HbE, resulted from the linked deletional α^+^-thalassemia. A similar phenomenon has been observed for Hb Q-Thailand, which has always been linked with the 4.2-kb deletional α^+^-thalassemia (-α^4.2^) [22]. Interestingly, although the C > A mutation of Hb Doi-Saket caused structural abnormality, clinical and red blood cell abnormalities were undetectable. Given that the Hb Doi-Saket mutation has always been linked to the -α^3.7^ deletion, coinheritance with deletional α-thalassemia 1 is capable of producing a HbH phenotype [23]. Since, genotypically, they show deletion of three α-globin genes (-α^Doi-Saket(3.7)^/–), it should be named Hb Doi-SaketH disease. The clinical features should be similar to that of Hb QH disease (-α^Q-Thailand(4.2)^/–) [24,25].

The amino acid substitution of Hb Doi-Saket was identical to that found at the same position in the α2-globin gene, which leads to Hb Zhaoqing [α2 9(A7)Asn > Lys, HBA2:c.30C > A], originally described in the city of Zhaoqing, Guangdong province in China. No data regarding this variant has been associated with deletional α^+^-thalassemia. Nevertheless, clinical silence has been reported in heterozygotes [18]. Different mutations occur on codon 9 of the α2-globin gene in Hb Park Ridge [α2 9(A7) Asn > Lys, HBA2:c.30C > G] and Hb Zurich-Hottingen [α2 9(A7) Asn > Ser HBA2:c.29A > G] [26,27]. Both variants show no functional abnormality or instability, and they are associated with normal hematological values. In addition, a mutation causing other Hb variants at the same position has been reported in the α1-globin gene, including Hb Delfzicht [α1 9(A7) Asn > Lys, HBA1:c.30C > G], which was observed in a 69-year-old Dutch female [28]; Hb Farnborough [α1 9(A7) Asn > Asp, HBA1:c.28A > G], which was found to be associated with an α^3.7^ mutation with British origin [29]; and Hb Anadour [α1 9(A7) Asn > Ser, HBA1:c.29A > G], which was originally found in Morocco [30]. The hematological parameters of these variants showed slight microcytosis and the absence of pathological consequences. Their hematological data were normal in the simple heterozygote. These mutations arising on both the α1- and α2-globin genes did not affect the stability and function of Hb.

In this study, we observed simple heterozygosity for Hb Doi-Saket in the proband and his father, while the son coinherited HbE along with Hb Doi-Saket. The interaction of these two Hb variants observed in his son could lead to another abnormal Hb. In the heterozygous Hb Doi-Saket, Hb Doi-Saket tetramer was assembed from the α^Doi-Saket^ and β^A^ chains, and Hb A_2_Doi-Saket is tetramerized from the α^Doi-Saket^ and δ^A^ chains. These two variants showed marked changes in the net charge of the tetramer owing to an additional positive charge of the lysine residue at the side chain of the α-chain per molecule. Consequently, these two variants eluted slower or had more sluggish mobility than that of HbA and HbA_2_. According to our reference laboratory and literature, the mobility pattern of Hb Doi-Saket was most consistently observed for the heterozygous Hb Q-Thailand. This might result in an incorrectly presumptive diagnosis of heterozygosity of Hb Q-Thailand [31].

In cation-exchange HPLC using the VARIANT II system, Hb Doi-Saket was eluted partially overlapping with HbA_2_, while Hb A_2_-Doi-Saket was distinctly separated and took longer retention time than that of HbA_2_. This Hb pattern was different from that observed for Hb Q-Thailand. Therefore, HPLC separated this variant more efficiently than CE did, and presumptive diagnosis was improved. The percentage of Hb Doi-Saket (Hb Doi-Saket + HbA_2_) using the HPLC system did not differ from that obtained using CE. Hb analysis using Premier Resolution HPLC showed a similar chromatogram as compared to those analyzed using VARIANT II HPLC. Hb analysis of heterozygous Hb Q-Thailand using our method revealed a different chromatogram from of Hb Doi-Saket. Markedly, analysis of Hb using both CE and HPLC could not accurately estimate Hb Doi-Saket, since it was concealed within HbF when analyzed by CE and HbA_2_ when analyzed by HPLC.

Interestingly, we demonstrated that the interaction of Hb Doi-Saket with HbE observed in the proband’s son caused complex thalassemia syndrome. Furthermore, it led to another novel variant resulting from the tetrameric assembly of α^Doi-Saket^ with the β^E^ globin chains, namely Hb EDoi-Saket (α^Doi-Saket^_2_β^E^_2_). We noted that the combined levels of HbA_2_ and HbE, and Hb Doi-Saket levels were lower than those estimated by CE. This observed reduction in the levels of combined HbA_2_ and HbE, and Hb Doi-Saket could be owing to the nonblack area under the peak of HbA_2_ and HbE, and Hb Doi-Saket representing in the inverted V shape not being integrated into total hemoglobin. In addition, Hb EDoi-Saket was coeluted with Hb A_2_-Doi-Saket. Interestingly, repetition of Hb analysis using another HPLC device could partially separate these two derivatives. This indicates that the Premier Resolution HPLC system is able to discriminate between the two new tetrameric variants, Hb EDoi-Saket (α^Doi-Saket^_2_β^E^_2_) and Hb A_2_-Doi-Saket (α^Doi-Saket^_2_δ^A^_2_), which were present in the son. In addition to these variants, Hb E and Hb Doi-Saket could be partially separated by this system. The proportion of HbE and Hb Doi-Saket were similar with those obtained by CE.

The level of circulating Hb Doi-Saket estimated in the simple heterozygotes (-α^Doi-Saket(3.7)^/αα, β^A^β^A^) with three α-globin genes was found to be approximately 32% of total Hb. This is in agreement with the levels of the α2α1 hybrid gene observed in Hb G-Philadelphia in *cis* to the -α^3.7^ deletion. The proportion of Hb G-Philadelphia with [-α^G-Philadelphia(3.7)^/αα, β^A^β^A^] genotype in peripheral blood was found to be approximately 33% [10,11,32], similar to that of Hb Doi-Saket. Similarly, the circulating level of the simple heterozygotes of the Hb Q-Thailand (-α^Q-Thailand(4.2)^/αα, β^A^/β^A^) ranged from 24–30% of the total Hb [4,14,22] and was similar to those observed in Hb Doi-Saket and Hb G-Philadelphia. This value is in agreement with the expression of the α1 gene. The α2 gene encodes proteins 2–3-folds more than does the α1 gene [33]; a relatively decreased transcription of the α2α1 hybrid gene, which was 65% of the expected value was owing to the loss of some sequences from 3′-end of the α2 gene [34].

The levels of Hb Doi-Saket observed in our two simple heterozygous conditions were relatively higher than that of Hb EDoi-Saket, noted as the coinheritance of Hb Doi-Saket and HbE (5.9%). The high level of Hb Doi-Saket compared to that of Hb EDoi-Saket indicates a higher affinity of the α^Doi-Saket^ chain for the β^A^ chain than for the β^E^ chain in producing Hb molecule. Furthermore, we observed no difference in the hematological values of the simple heterozygous Hb Doi-Saket (proband and his father) and double heterozygous Hb Doi-Saket and HbE (his son), though the son had an Hb level lower than those of the proband and his father. These results indicate that coexistence of Hb Doi-Saket with heterozygous HbE does not contribute further to the severity of anemia in the patient. This is the first report of Hb Doi-Saket migrating with the same electrophoretic mobility and showing same chromatographic retention as those of Hb Q-Thailand, an α1-Hb variant occasionally found in the Southeast Asian population [4,22]. These findings highlight and reinforce the importance of Hb analysis using more than one method, leading to the selection of an appropriate molecular examination to identify the variant.

The developed PCR–RFLP method was successfully used for the definitive diagnosis of Hb Doi-Saket, and the newly developed multiplex ASPCR method effectively and accurately detected Hb Doi-Saket and Hb Q-Thailand simultaneously in one experiment. The effectiveness of this multiplex ASPCR method should facilitate a prevention and control program of hemoglobinopathy in the region where thalassemia and hemoglobinopathies are prevalent with high heterogeneity.

Haplotypic expression of the six polymorphic sites of the α-globin gene cluster and one inter ζ-globin HVR region were characterized by the method described here. Fortunately, the family members of the proband participated, allowing us to segregate the haplotype accurately in all chromosomes. Hb Doi-Saket was linked to haplotype [- + M + + 0 -], which indicates the origin and evolution of the Hb Doi-Saket mutation in Thai ancestry; however, this haplotype seems to be different from that of Hb Q-Thailand that is an α-Hb variant linked with (-α^4.2^), which has been previously documented in Thai populations [+ nd *S* + 0 - -] [22]. These two variants might share a different origin. Nevertheless, the haplotype of Hb Doi-Saket linked to the α^3.7^ mutation, similar to that of the Thai -α^3.7^ allele [19], might indicate that they share the same origin. Nonetheless, information on Hb Zhaoqing, an identical α2 mutation at the same position as that of Hb Doi-Saket, was not available. The origin of Hb Zhaoqing mutation in Chinese populations could be same as that of Hb Doi-Saket.

## Conclusion

This study initially demonstrated a novel mutation, Hb Doi-Saket, on the α2α1 hybrid gene. This variant has always been linked to the -α^3.7^ deletion; individuals heterozygous for this variant may show slight microcytosis and might have severe clinical entities when it is associated with deletion in α-globin genes. The identification of Hb Doi-Saket in this study emphasizes that different separative methods can provide useful information in patient management and reinforces the importance of a complete molecular approach, even with a silent hematological phenotype.

## Data Availability

The data that support the findings of this study are available from the corresponding author upon reasonable request.
